# Challenging case of muscle bridge; a 15-year follow-up of a patient

**DOI:** 10.22088/cjim.11.1.120

**Published:** 2020

**Authors:** Hassan Aghajani, Kaveh Hosseini, Saeed Alizadeh, Reyhaneh Aghajani

**Affiliations:** 1 Department of Cardiology, Tehran Heart Center, Tehran University of Medical Sciences, Tehran, Iran; 2 Tehran Heart Center, Tehran University of Medical Sciences, Tehran, Iran; 3 School of Medicine, Tehran University of Medical Sciences, Tehran, Iran

**Keywords:** Refractory myocardial bridging, Percutaneous coronary intervention, Coronary artery bypass graft, In-stent restenosis, graft failure

## Abstract

**Background:**

Anatomically myocardial bridging (MB) consists of either superficial myocardial fibers that traverse over the LAD or deep fibers that encircle the coronary artery. In this study, we present a patient with myocardial bridging, who was primarily diagnosed with coronary artery disease which did not properly respond to full-dose medical treatment but benefited from coronary artery bypass graft (CABG).

**Case presentation:**

In 2017, a 53-year old man was referred to Tehran Heart Center (THC) with complaint of typical chest pain (TCP). In 2003 he had TCP and underwent coronary angiogram (CAG), due to positive non-invasive tests. Muscle-bridge in LAD was diagnosed. In 2007, he was symptomatic and another CAG was done, and percutaneous coronary intervention (PCI) with stenting was performed. In 2008 he became symptomatic and his interventionist, decided to perform another CAG. At that time, he had CABG. He was asymptomatic until 2015, he referred to us with the same TCP and we decided to perform CAG for the fourth time. After two years, again another PCI was done due to in-stent restenosis.

**Conclusion:**

Revascularization should be considered in MB refractory to medical treatment. However, coronary perforation, in-stent restenosis and graft failure are major concerns.

Myocardial bridging is a congenital coronary anomaly in which a segment of an epicardial coronary artery- the tunneled artery- (most frequently the middle segment of the left anterior descending artery) takes an intramuscular course.(1) This condition causes a temporary systolic coronary arterial luminal narrowing. Incidence of myocardial bridging varies from 1.5% to 16%.(2) Myocardial bridging is often asymptomatic, however, it may lead to complications such as angina, coronary spasm, myocardial ischemia and acute coronary syndromes, arrhythmias, and rarely sudden cardiac death.(3, 4). In this study, we present a patient with myocardial bridging who underwent revascularization several times. Findings of the current report emphasize on the importance of considering CABG/PCI in patients who are refractory to medical managements. However, this invasive approach have many complications; perforation, in stent restenosis, stent fracture and graft failure. Here we discuss a 15-year follow-up of our patient with almost all of the mentioned adverse events. 

## Case presentation

In 2017, a 53-year old man was referred to Tehran Heart Center (THC) with chief complaint of typical chest pain (TCP) and positive myocardial perfusion scan test. 

**Figure 1:**
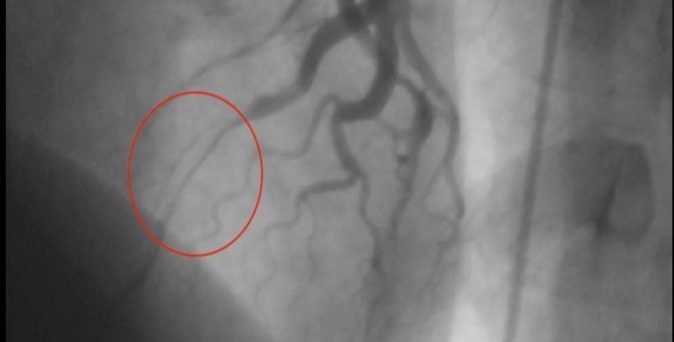
Muscle-bridge in LAD

His past medical history was as follows; in 2003 he had TCP and underwent coronary angiogram (CAG) in Hamedan due to positive non-invasive tests. ECG at that time was unremarkable. Muscle-bridge in LAD was diagnosed (video 1 and figure 1). In 2007, he became symptomatic despite full-dose medical treatment (propranolol and diltiazem at maximum tolerable doses) and non-invasive testing (myocardial perfusion imaging [MPI]) was also positive. Hence another CAG was done at THC, and percutaneous coronary intervention (PCI) with stenting was performed, Taxus 2.75-32 @ 18 atm was implanted (video 2). Micro perforation happened during stent deployment, (video 3) a balloon was inflated at site for 10 minutes to stop the leakage. Overlap stenting was also done for another stenosis using Taxus 3-16 @ 16 atm. The patient was symptom free for about a year, however in 2008, he became symptomatic and his local interventionist in Hamedan Hospital decided to perform another CAG (video 4). 

**Figure 2:**
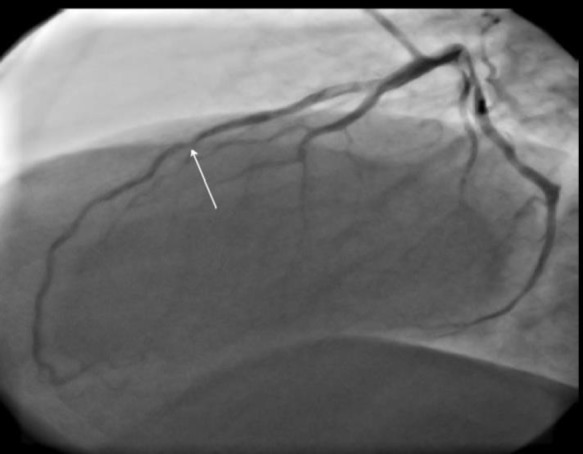
In-stent restenosis in proximal part of previous stent in LAD

**Figure 3:**
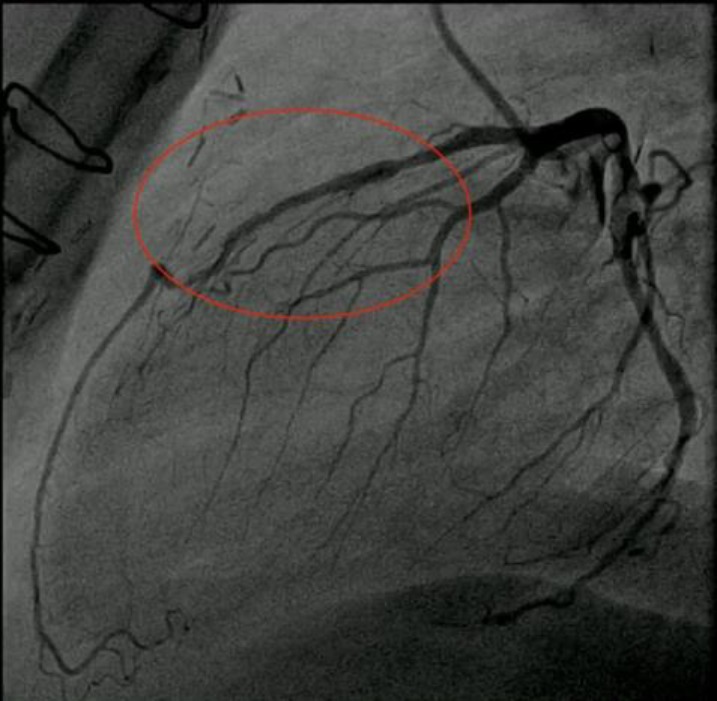
In- Stent restenosis of LAD

In-stent restenosis was seen in proximal part of previous stent (figure 2). At that time, he underwent coronary artery bypass graft surgery (CABG) with LIMA graft on LAD and myotomy. He was asymptomatic for just a few months and again he experienced the same typical chest pain and thus we decided to perform CAG for the fourth time (video 5). Diffuse proliferative in-stent restenosis was seen, (figure 3) and left internal mammary artery (LIMA) was non-functional (video 6). Plain old balloon angioplasty (POBA) was done on LAD and drug eluting balloon was deployed and inflated. Stent was deployed for distal lesion (Orsiro (BIOTRONIK Inc. Berlin, Germany) 2.5-15 @ 8 atm) (video 7). He was asymptomatic for two years, but again same scenario occured in 2017. Another PCI was done due to significant proximal LAD lesion (video 8). Acceptable POBA on LAD was done (video 9).

## Discussion

Myocardial bridging is a common finding at autopsy of normal patients and therefore it had been thought to be a benign anatomic variation (1). Typical myocardial bridge can range from 4 to 80 mm in length and from 0.3 to 28 mm in depth (5). Myocardial bridges of the LAD artery have been categorized into two types. The more common “superficial” bridges (approximately 75% of patients) cross over the LAD and “deep” variants in which the LAD artery is encircled by bridging fibers (5). The prevalence of myocardial bridging differs based on the mode of evaluation. A mean frequency of myocardial bridging of 25 percent (range 5 to 86 percent) have been observed in pathologic studies and non-invasive methods, similar results have been reported in noninvasive imaging studies using coronary computed tomography (3). 

Angiographic studies which shows systolic compression, have noted different findings. The reported prevalence of myocardial bridging among patients undergoing coronary angiography, is about 1.7 percent, although this number can increase up to 40% if provocation tests are used (2, 3). While left anterior descending artery is the most commonly affected artery (67-98%), right coronary artery (RCA), left circumflex (LCX) artery, diagonal (18%) and marginal (40%) branches are also commonly involved as well (5). 

Angiographic significance depends on multiple factors including the thickness and the length of the bridged segment, orientation of the coronary artery to the myocardial fibers, coronary smooth muscle tone, the presence of loose connective or adipose tissue around the bridged segment, myocardial contractility, the nature of the tissue interposed between the coronary artery and the myocardium, and observer experience (1). That partially explains why many patients with myocardial bridging are considered normal coronary on angiography.

Based on autopsies and intravascular ultrasounds (IVUS), distal segments of bridged vessels remain free from atherosclerosis while the proximal segments are susceptible to atherosclerosis (3, 5). First-line therapy for patients with myocardial bridging consists of beta-blockers and non-dihydropyridine calcium-channel blockers (3, 5) while nitrates are contraindicated in patients with myocardial bridging (3). Percutaneous coronary intervention (PCI) can normalize flow and abolish symptoms in these patients (3, 6). Stent fracture, coronary perforation during stent deployment, in-stent restenosis and stent thrombosis are the most common complications (5, 7). Surgical options for myocardial bridging include surgical myotomy and coronary artery bypass graft surgery (CABG). Studies on myotomy have shown overall successful results. Accidental right ventricular wall perforation has been reported as a complication of surgical procedures (5). Coronary artery bypass grafting is an acceptable treatment option for myocardial bridging. CABG can be helpful particularly in patients with deep bridges (>5 mm) (3, 5). In a study of 39 patients with muscle bridging who had undergone CABG, graft occlusions occurred in 15 out of 39 patients in follow up period (8). 

Grafting with the LIMA was more likely to result in occlusion compared to grafting with the saphenous vein. Limited data suggest that surgical therapy, either myotomy or CABG, appears safe and effective in symptomatic patients with myocardial bridging who experience refractory episodes of chest pain despite maximum medical therapy (5, 9). It has been suggested that surgical intervention might be a better choice than coronary stenting in myocardial bridging (4, 6). 

In conclusion**, **traditionally, myocardial bridges have been considered to be a benign condition, but several recent studies have demonstrated that their clinical complications can be dangerous and prognosis might not be as good as once was thought. Myocardial bridging may predispose to coronary vasospasm which leads to ischemic episodes. Thus, revascularization (surgical intervention, or less preferably PCI) (10) should be considered in myocardial bridging refractory to medical treatment. However, coronary perforation, in-stent restenosis and graft failure are major concerns.

## Funding:

 This study was not funded. 

## Conflicts of Interest:

Authors declare no Conflict of Interests for this article.
